# Differential Functions of Pepper Stress-Associated Proteins in Response to Abiotic Stresses

**DOI:** 10.3389/fpls.2021.756068

**Published:** 2021-12-10

**Authors:** Yeongil Bae, Chae Woo Lim, Sung Chul Lee

**Affiliations:** Department of Life Science (BK21 Program), Chung-Ang University, Seoul, South Korea

**Keywords:** ABA, drought, low temperature, pepper, virus-induced gene silencing

## Abstract

Stress-associated proteins (SAPs), a group of zinc-finger-type proteins, have been identified as novel regulators of plant abiotic and biotic stresses. However, although they have been discovered in different plant species, their precise functional roles remain unclear. Here, we identified 14 *SAP* subfamily genes in the pepper genome. An investigation of the promoter regions of these genes for *cis*-regulatory elements associated with abiotic stress responses revealed the presence of multiple stress-related elements. Domain and phylogenetic analyses using the corresponding protein sequences revealed that the *CaSAP* genes can be classified into six groups (I–VI) and sorted into two broad types. Expression levels of the *CaSAP* genes were found to be differentially induced by low temperature, the dehydration stress, or exogenous abscisic acid. Group II and IV genes were highly induced by the low temperature and dehydration treatments, respectively. Moreover, subcellular localization analysis indicated that the proteins in these two groups are distributed in the nucleus, cytoplasm, and plasma membrane. Among the pepper plants silenced with the three identified group II *CaSAP* genes, the *CA02g10410*-silenced plants showed tolerance to low temperature, whereas the *CA03g17080*-silenced plants were found to have temperature-sensitive phenotypes. Interestingly, group IV *CaSAP*-silenced pepper plants showed drought-tolerant phenotypes. These findings contribute to a preliminary characterization of *CaSAP* genes and provide directions for future research on the biological role of *CaSAPs* in response to different abiotic stresses.

## Introduction

With the ever-increasing severity of environmental problems, plants are becoming more frequently subjected to adverse abiotic stresses, such as extreme temperatures, high salinity, and dehydration, which contribute to limiting plant growth and crop productivity (Hasegawa et al., 2000; Park et al., 2016; He et al., 2018; Waqas et al., 2019). To survive under stress conditions, plants accordingly need to adapt to these stresses by mounting appropriate defense responses. In this regard, signaling molecules, such as different types of protein kinases, protein phosphatases, transcription factors, and other regulatory proteins, participate in several stress signaling pathways to maintain homeostasis (Shinozaki and Yamaguchi-Shinozaki, 2000; Schroeder et al., 2001; Zhu, 2002; Ding et al., 2020). In response to the transduction of such signals, downstream target genes are up- or downregulated (Chinnusamy et al., 2005; Tuteja, 2007; Ding et al., 2020), thereby enabling plants to adaptively modify their physiological or morphological status (Parihar et al., 2015; Volkov, 2015; Liu et al., 2018).

Exposure to the low temperature stress, also referred to as cold stress, can enable plants to cold acclimate under temperate climatic conditions (Guo et al., 2018); it can be divided into chilling (0–15°C) and freezing (under 0°C) stresses. Chilling stress is the primary stress to which subtropical crops, such as rice, are subjected; to adapt to chilling stress conditions, plants have evolved the ability to perceive cold signals and the transduction of related signals. In this context, the C-repeat (CRT)-binding factors/dehydration-responsive element binding protein 1 (CBF/DREB1)-dependent signaling pathway has been established to be the main regulatory mechanism activated in response to the low temperature stress (Thomashow, 1999). Recent studies have reported that a range of different proteins, including kinases, transcription factors, and regulator proteins, are involved in this mechanism, which leads to the activation of cold-regulated (*COR*) genes and enhanced chilling tolerance (Shi et al., 2018; Ding et al., 2020). Moreover, water deficit and elevated temperature are severe issues that contribute to promoting the dehydration stress in plants (Park et al., 2016; Lim et al., 2018a; Zhang et al., 2021). In this regard, plants can alter their physiological or morphological patterns by controlling changes in the balance of certain hormones (Waqas et al., 2019), among which the phytohormone abscisic acid (ABA) is known to play key roles in the responses to the dehydration stress (Cutler et al., 2010). The main ABA signaling pathway is the PYR/PYL/RCAR-PP2Cs-SnRK2s cascade (Shinozaki and Yamaguchi-Shinozaki, 2000; Nakashima and Yamaguchi-Shinozaki, 2013; Sah et al., 2016). Nevertheless, although the responses of plants to abiotic stress have been generally well characterized, much is still unknown regarding the underlying mechanisms, and studies on stress responses are accordingly still being actively conducted.

Stress-associated proteins (SAPs) are a group of zinc-finger-type proteins that are reportedly associated with abiotic stress responses, immunity, and development (Mukhopadhyay et al., 2004; Dixit et al., 2018; Dong et al., 2018; Liu et al., 2019c; Wang et al., 2019; Zhao et al., 2020). Since the discovery of the first *SAP* gene, the *OsiSAP1* (*OsSAP1*) gene in rice, others have been characterized in various plant species as genome sequencing technology has advanced (Mukhopadhyay et al., 2004; Vij and Tyagi, 2006; Solanke et al., 2009; Giri et al., 2013; Dong et al., 2018; Liu et al., 2019a; Zhang et al., 2019; Ben Saad et al., 2020; Lai et al., 2020; Wang et al., 2021). However, no *SAP* genes have been isolated from pepper plant. Relatively well-characterized SAPs have been found to contain an A20 domain at the N terminus and/or an AN1 domain at the C terminus, which are highly conserved across species (Vij and Tyagi, 2008; Lai et al., 2020). In animals, the A20 zinc-finger proteins function as negative regulators of inflammation and have de-ubiquitinating activity (Lademann et al., 2001; Evans et al., 2004). Although the precise function of the AN1 zinc-finger proteins is unclear, recent studies have reported that they may regulate protein–protein interactions (Linnen et al., 1993; Chang et al., 2011). In plants, several SAPs containing A20/AN1 zinc-finger domains have been reported to confer tolerance to multiple abiotic stresses (Huang et al., 2008; Kanneganti and Gupta, 2008; Giri et al., 2011; Lloret et al., 2017; Yoon et al., 2018; He et al., 2019) and play a role in immunity (Liu et al., 2019b,c). Moreover, *Arabidopsis* SAP5 has been reported to have E3 ubiquitin ligase activity (Kang et al., 2011). A further type of SAP consists of an N-terminal AN1 zinc-finger domain and/or a C-terminal Cys2-His2 (C2H2) zinc-finger domain, and over the past two decades, C2H2 zinc-finger proteins have been studied and reported to have diverse functions in plant growth, development, and biotic/abiotic stress resistance (Han et al., 2020).

Pepper (*Capsicum annuum*) is one of the most important vegetable crops cultivated globally that has economic value as a spice, medicine, vegetable, and biopesticide (Lim et al., 2018b). Although the demand for peppers is increasing worldwide, their productivity can be limited to varying extents by adverse environmental conditions, such as dehydration, high salinity, and extreme temperatures. To solve this problem, numerous studies have focused on the defense mechanisms activated in response to such environmental stresses (Chen et al., 2014; Guo et al., 2014; Park et al., 2016; Lim et al., 2018a,^2020^; Kang et al., 2020; Wu et al., 2020). In recent decades, numerous stress-related genes have been discovered in the pepper plants. Furthermore, the recent whole-genome sequencing of pepper has accelerated these ongoing research efforts (Kim et al., 2014; Qin et al., 2014; Hulse-Kemp et al., 2018). Nonetheless, to our knowledge, *SAP* gene family and their functional role, especially in response to dehydration and low temperature, have been not yet identified from pepper plants.

In the present study, we performed a genome-wide analysis of *SAP* family genes in *C. annuum* and identified 14 *SAP* genes, the expression patterns of which were investigated in response to different abiotic stresses and exogenous ABA. In addition, we selected six *SAP* genes for conducting phenotypic assays. It is anticipated that the findings of this study will make a significant contribution to advancing our current understanding of plant SAPs and the mechanisms underlying the responses of plants to abiotic stress.

## Materials and Methods

### Plant Material and Growth Conditions

In the present study, we used pepper (*Capsicum annuum* cv. Nockwang) and tobacco (*Nicotiana benthamiana*) as experimental plants. Pepper seeds were soaked in a growth chamber at 28°C under dark conditions for 4 days. The germinated seeds were then planted in a mixture of steam-sterilized soil (peat moss, perlite, and vermiculite, 5:3:2, v/v/v), sand, and loam soil (1:1:1, v/v/v). The seeds of tobacco plants were sown in the same soil mixture. Both plant types were grown in a growth room at 25 ± 1°C and 60% relative humidity under white fluorescent light (130 μmol photons⋅m^–2^⋅s^–1^) on a 16 h light/8 h dark cycle.

### Abiotic Stress Treatments

Plants were subjected to different abiotic stresses, the effects of which were subsequently assessed based on quantitative reverse transcription-polymerase chain reaction (qRT-PCR) and phenotypic analyses. Pepper plants were subjected to dehydration by detaching the shoots and subsequently harvesting the leaves at designated time points (0, 6, and 12 h). For low temperature treatments, pepper plants were placed in an unilluminated growth chamber at 10°C, and leaves were harvested after 0, 6, and 12 h. Other plants were treated with ABA (100 μM) or mannitol (600 mM), with leaves again being collected after 0, 6, and 12 h as described by previous studies (Lim et al., 2019; Lim C. W. et al., 2021; Lim J. et al., 2021).

### RNA Isolation and Quantitative Reverse Transcription-Polymerase Chain Reaction

Total RNA was isolated from the leaves of pepper plants at the 6th-leaf stage. The RNA thus obtained was quantified using spectrophotometer, and 1 (μg of the quantified RNA was used as a template to synthesize cDNA using the iScriptTM cDNA synthesis kit (Bio-Rad, Hercules, CAalifornia, United StatesA). The synthesized cDNA was amplified in a CFX96 TouchTM Real-Time PCR detection system (Bio-Rad) using the iQTMSYBR Green Supermix (Bio-Rad) and specific primers (Supplementary Table 1). Pepper *Actin1* (*CaACT1* and *CA12g08730*) was used as an internal control.

### Subcellular Localization

The coding regions of the *CaSAP* genes (groups II and IV), minus stop codons, were inserted into a bar 35S-GFP vector containing the cauliflower mosaic virus 35S promoter and a green fluorescent protein (GFP) tag at the C-terminal end of the insert. *Agrobacterium tumefaciens* strain GV3101 harboring the GFP-tagged *CaSAP* (group II and IV) constructs was mixed with *Agrobacterium* strain p19 (1:1; OD_600_ = 0.5) and co-infiltrated into the epidermal cells of 5-week-old *Nicotiana benthamiana* leaves to induce transient expression. The 35S-GFP empty vector was used as a positive control. Two days after infiltration, we examined GFP signals using an LSM700 confocal microscope (Carl Zeiss, Jena, Germany) and analyzed the signals using the ZEN 3.1 software. DAPI (1 μg/ml) was used as a nucleus marker and FM4-64 (50 μM) as a plasma membrane marker.

### A Virus-Induced Gene Silencing System

A tobacco rattle virus (TRV)-based virus-induced gene silencing system was used to generate a *CaSAP* (group II and IV) knock-down model in pepper plants, as described in previous studies (Pflieger et al., 2013; Jeong et al., 2020). We designed 300 bp fragments for each cDNA of *CaSAP* (groups II and IV), which were validated as regions for gene silencing using the VIGS tool^1^. *Agrobacterium tumefaciens* strain GV3101 carrying pTRV1 and pTRV2:*CaSAP* (groups II and IV) or pTRV2:00 (negative control) was co-infiltrated into the cotyledons of 2-week-old pepper plants (OD_600_ = 0.2 for each construct). The infected pepper plants were cultivated under the aforementioned growth conditions, and 2 weeks later, we measured the expression of each *CaSAP* gene using qRT-PCR (Supplementary Figure 1).

## Results

### Identification of *SAP* Family Genes in *Capsicum annuum*

**FIGURE 1:**
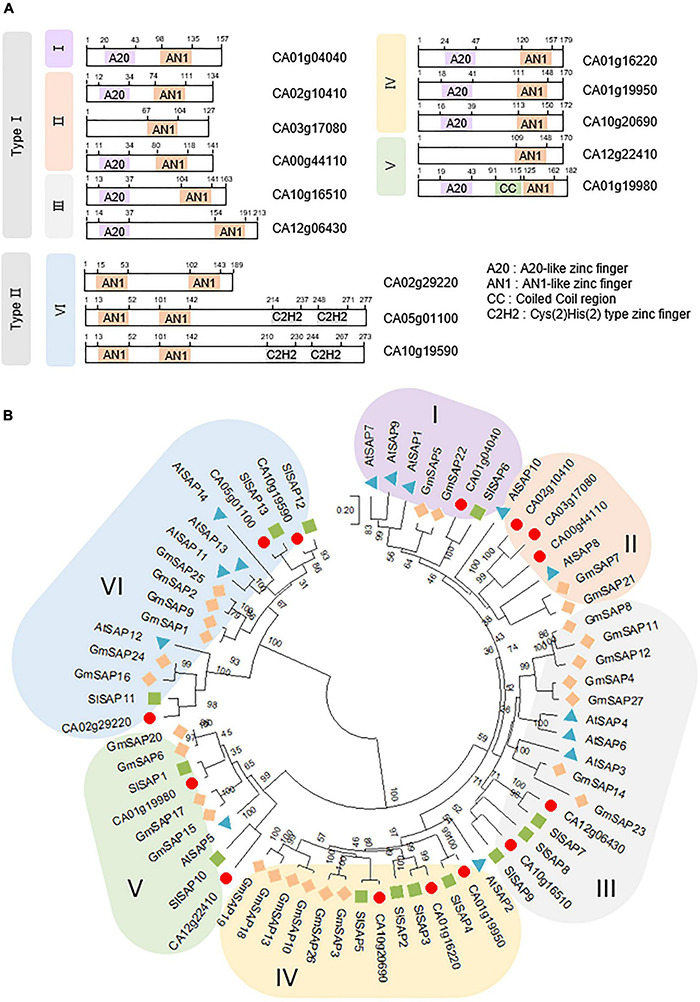
Pepper stress-associated protein (*SAP*) subfamily genes. **(A)** Domain organization of the pepper *SAP* subfamily genes. The amino acid sequences were obtained from SGN (https://solgenomics.net/), and domains were predicted using SMART (Simple Modular Architecture Research Tool; http://smart.embl-heidelberg.de/). **(B)** Phylogenetic tree analysis of the pepper *SAP* subfamily genes. The amino acid sequences were deduced from the full-length coding sequences of *SAP* genes in pepper (red circles), *Arabidopsis* (blue triangles), soybean (orange diamonds), and tomato (green squares) and used for comparisons. The phylogenetic tree was generated based on the neighbor-joining method implemented in MEGA X software.21. Bootstrap values shown at branch points were calculated from 1,000 bootstrap replications. The scale bar denotes the evolutionary distance computed using the Poisson correction method.

In recent decades, *SAP* family genes have been identified as novel regulators involved in the responses of plants to different stresses, such as drought, high salinity, and extreme temperatures; additionally, they are known to play crucial roles in plant immunity (Mukhopadhyay et al., 2004; Kanneganti and Gupta, 2008; Ströher et al., 2009; Dixit and Dhankher, 2011; Tyagi et al., 2014; Liu et al., 2019b). To investigate whether members of the SAP family are present in pepper and function similarly to those of other species, we obtained the amino acid sequences of 14, 27, and 13 *SAP* family genes from *Arabidopsis*, soybean, and tomato, respectively, and then searched for pepper *SAP* family genes using these sequences as a query in BLASTP searches. We identified 14 *SAP* genes in pepper containing the typical *SAP* conserved domains, including the AN1, A20, and C2H2 zinc-finger domains (Table 1 and Figure 1A). On the basis of the location of the AN1 domain (the N or C terminus), the 14 pepper *SAP* genes can be divided into two types (Type I and II, respectively). Moreover, the predicted subcellular localization indicated that these genes are primarily distributed in the nucleus (Table 1). Using the amino acid sequences of *SAP* genes in pepper, *Arabidopsis*, soybean, and tomato, we conducted phylogenetic analysis based on the neighbor-joining method implemented using the MEGA X software (Kumar et al., 2018). We found that the pepper *SAP* genes were clustered into six clades (designated groups I to VI), each of which contained one to three members of the 14 pepper genes (Figure 1B). As several previous studies have reported that the functions of SAP proteins are primarily associated with responses to abiotic stresses, we focused on these responses in the present study.

**TABLE 1:** Information on the *CaSAP* subfamily genes.

Group	Gene locus	Chromosome	Location	Strand direction	CDS (bp)	Intron	Predicted protein (aa)	pI	MW (kDa)	Zing finger domain	Predicted subcellular location
I	CA01g04040	01	6602752–6603225	–	474	0	157	8.59	16.91	A20-AN1	Chloroplast, Nucleus, Cytoplasm
II	CA02g10410	02	129918896–129919300	–	405	0	134	8.88	15.28	A20-AN1	Nucleus, Chloroplast
	CA03g17080	03	196705800–196706183	–	384	0	127	7.95	14.21	AN1	Chloroplast, Nucleus, Cytoplasm
	CA00g44110	nd	nd		426	0	141	8.88	15.76	A20-AN1	Nucleus
III	CA10g16510	10	220169266–220169757	–	492	0	163	7.53	18.09	A20-AN1	Chloroplast
	CA12g06430	12	22845946–22846587	+	642	0	213	8.25	23.52	A20-AN1	Mitochondria, Chloroplast, Cytosol
IV	CA01g16220	01	101708816–101709355	+	540	0	179	7.94	19.16	A20-AN1	Chloroplast
	CA01g19950	01	160637657–160638169	+	513	0	170	8.43	18.33	A20-AN1	Chloroplast
	CA10g20690	10	230827282–230827800	+	519	0	172	8.62	18.23	A20-AN1	Nucleus
V	CA01g19980	01	160706592–160707140	+	555	0	184	9.12	20.26	A20-AN1	Nucleus
	CA12g22410	12	234718042–234718554	–	513	0	170	9.25	19.19	AN1	Nucleus
VI	CA02g29220	02	168266440–168267317	–	570	1	189	8.96	20.81	AN1-AN1	Nucleus
	CA05g01100	05	1965718–1969104	–	834	1	277	8.70	31.25	AN1-AN1-C2H2-C2H2	Chloroplast, Nucleus
	CA10g19590	10	228970650–228973903	+	822	1	273	8.79	30.28	AN1-AN1-C2H2-C2H2	Nucleus

*Gene locus and location are from Capsicum annuum cv ‘CM334’ genome (release 1.55). Domain analysis is performed using a web tool SMART (Simple Modular Architecture Research Tool; http://smart.embl.de/) Subcellular localization of protein is predicted using a web tool WOLF PSORT (http://www.genscript.com/wolf-psort.html). nd, not detected due to unsequenced portion of gene.*

### Expression Patterns of the Pepper SAP Family

**FIGURE 2:**
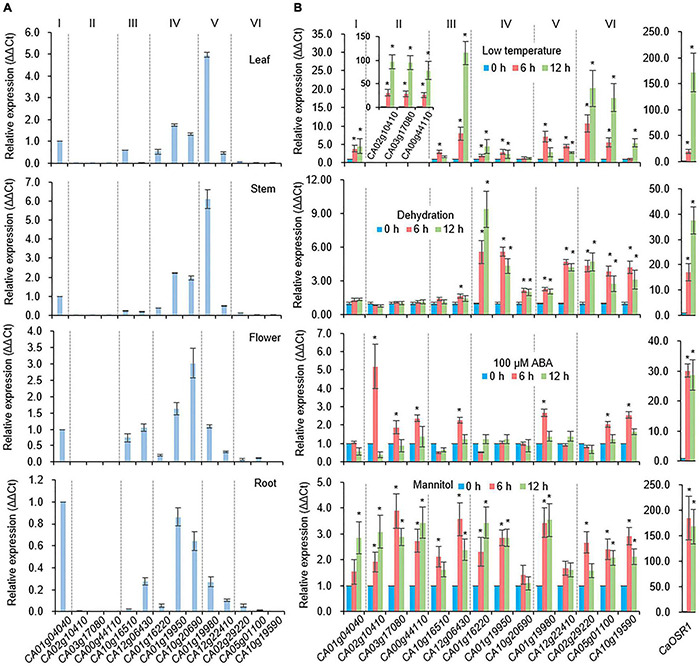
Expression patterns of *CaSAP* genes. **(A)** Tissue-specific expression of *CaSAP* genes. The expression levels of *CaSAP* genes were determined via qRT-PCR analysis using cDNA derived from the first- and second-leaves, stems, and roots harvested from the six-leaf stage pepper plants and fully opened flowers. Values for *CA01g04040* were set to 1.0. **(B)** Expression levels of *CaSAP* genes in response to different abiotic stresses. Expression levels of *CaSAP* genes were determined via qRT-PCR analysis using cDNA derived from the first and second leaves of pepper plants (six-leaf stage) subjected to different abiotic stresses: dehydration (shoot detachment), ABA (100 μM), mannitol (600 mM), and low temperature (10°C). The relative expression (ΔΔCT) of *CaSAP* genes was normalized to that of *CaACT1*, which was used as an internal control gene, and values at 0 h were set to 1.0. Values are presented as the mean ± standard error of values from three independent experiments. Asterisks indicate significant differences compared to the value at 0 h for each gene (Student’s *t*-test; *P < 0.05).

To investigate the tissue-specific expression of pepper *SAP* genes, we conducted qRT-PCR using cDNA templates derived from different tissues (leaf, stem, flower, and root) (Figure 2A). In common, group II and VI *CaSAP* genes showed low expression levels in all tissues. The expression level of group V *CA01g19980* was higher in the leaf and stem than other *CaSAPs*. In the flower, the transcript of group IV *CA10g20690* showed the higher level than that of other *CaSAPs*. Under water deficit conditions, dehydration is typically caused, which enhances biosynthesis and accumulation of the phytohormone ABA as signal molecules (Cutler et al., 2010). Cold stress also leads to dehydration in plant by reducing root water uptake (Steponkus, 1984). Based on these, we initially analyzed whether proteins in the pepper SAP family (CaSAP) play functional roles in response to water stress caused by dehydration and low temperature. First, we performed qRT-PCR using template cDNA obtained from plants that had been subjected to different stress treatments (low temperature, dehydration, ABA, and mannitol), and the primers listed in Supplementary Table 1. As a positive control for stress treatment, we used *CaOSR1*, which is homologous to *Arabidopsis RD29B* (Park et al., 2016; Lim et al., 2020). It is noteworthy that members of the aforementioned six groups tended to show differential responses to different stresses (Figure 2B). For example, after 6 and 12 h of treatment, the expression levels of group II *CaSAP* genes were found to increase more prominently in response to low temperature (10°C) than those of the other groups. Similarly, we detected significant increase in the expression levels of group IV, V, and VI *CaSAP* genes in plants subjected to the dehydration stress. In particular, compared with the other *CaSAP* family genes, *CA01g16220*, a group IV *CaSAP* gene, showed the highest level of expression at 12 h after dehydration treatment. In response to mannitol treatment, most of the *CaSAP* genes showed significantly increased expression compared with that in the control. ABA is a key phytohormone that regulates abiotic stress responses (Hasegawa et al., 2000; Shinozaki and Yamaguchi-Shinozaki, 2000; Schroeder et al., 2001); hence, we sought to determine whether expression of the *CaSAP* genes shows differential responses to ABA. Six hours after ABA treatment, we detected the upregulated expression of seven of the 14 *CaSAP* genes. Subsequently, we analyzed the promoter region *cis*-regulatory elements to gain an understanding of stress- and ABA-induced transcriptional regulation of the *CaSAP* genes (Table 2). Specifically, we obtained 3 kb upstream sequences of the *CaSAP* genes from the *Capsicum annuum* cv. CM334 (Criollo de Morelos 334) genome database accessed from SGN^2^ and used the New PLACE web tool^3^ to predict the *cis*-regulatory elements. We accordingly identified a number of stress- and ABA-responsive *cis*-elements, including stress response element (STRE), low temperature-responsive element (LTR), dehydration-responsive element (DRE), TC-rich repeats, ABA-responsive element (ABRE), MYB, MYC, and MBS (Narusaka et al., 2003; Yamaguchi-Shinozaki and Shinozaki, 2005; Table 2). A majority of the *CaSAP* genes were found to contain an abundance of ABRE, MYB, MBS, and MYC motifs, whereas TC-rich repeats, DRE1, and DRE core were rarely predicted in these genes. Furthermore, with the exception of group VI *CaSAP* genes, STRE was detected in abundance. These findings provide evidence to indicate that members of the CaSAP family are regulated by different *cis*-regulatory elements, resulting in transcriptional regulation in response to multiple abiotic stresses.

**TABLE 2:** *Cis*-regulatory elements information of *CaSAP* subfamily. Gene locus are from *Capsicum annuum* cv. ‘CM334’ genome (release 1.55).

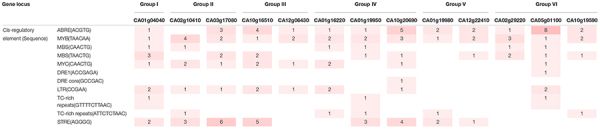

### Subcellular Localization of CaSAPs

**FIGURE 3:**
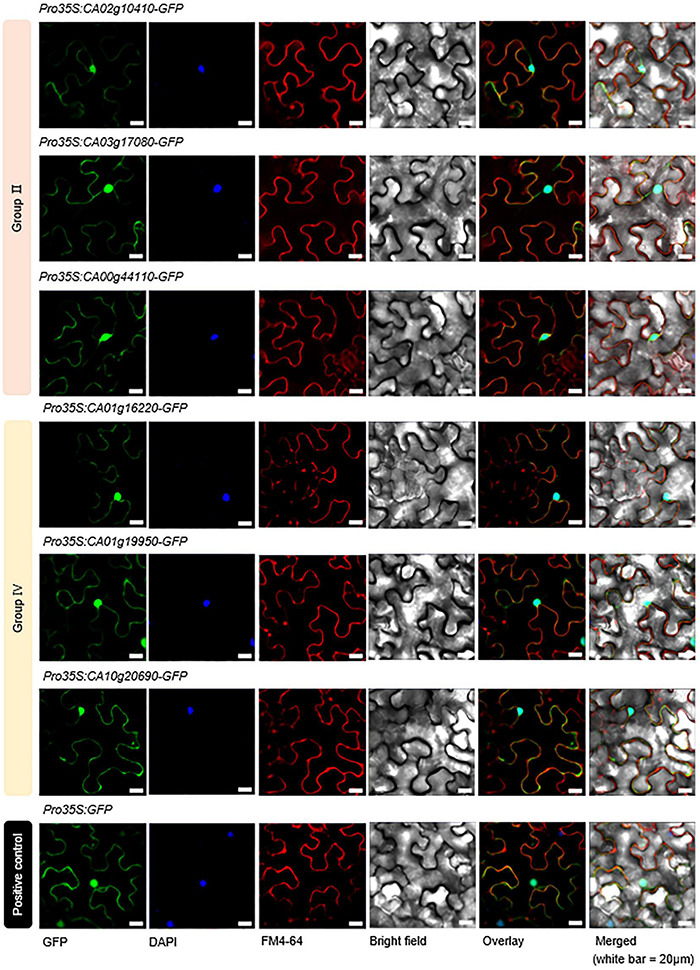
Subcellular localization of CaSAP proteins. The leaves of *Nicotiana benthamiana* were transformed with constructs harboring SAP–GFP fusion proteins via *Agrobacterium*-mediated infiltration. Confocal images show transiently transformed *N. benthamiana* epidermal cells expressing group II (CA02g10410, CA03g17080, and CA00g44110) and group IV (CA01g16220, CA01g19950, and CA10g20690) SAP–GFP fusion proteins. The 35S-GFP empty vector was used as a positive control. White bar = 20 μm.

In previous studies on rice, *Arabidopsis*, soybean, and tomato, most of the SAP family proteins have been detected in the nucleus and cytoplasm (Kothari et al., 2016; Dixit et al., 2018; Zhang et al., 2019; Zhao et al., 2020). In the present study, we similarly predicted that these proteins would show nuclear and cytoplasmic distribution in pepper (Table 1). On the basis of these predictions, we performed subcellular localization experiments for six of the identified CaSAP proteins (CA02g10410, CA03g17080, CA00g44110, CA01g16220, CA01g19950, and CA10g20690) tagged with green fluorescent protein (Figure 3). We designed *A. tumefaciens* strain GV3101 constructs harboring the *p35S*-*CaSAPs*-*GFP* vector, and transiently expressed the fusion proteins in leaf epidermal cells of *N. benthamiana*. Consistent with the findings obtained for other plants, we found that the CaSAP proteins were distinctly localized in the nucleus, cytoplasm, and plasma membrane (Figure 3), which implied that these six CaSAPs are functionally active in the pepper cell.

### Group II *CaSAP*-Silenced Pepper Plants Show Altered Phenotypes in Response to the Low Temperature Stress

**FIGURE 4:**
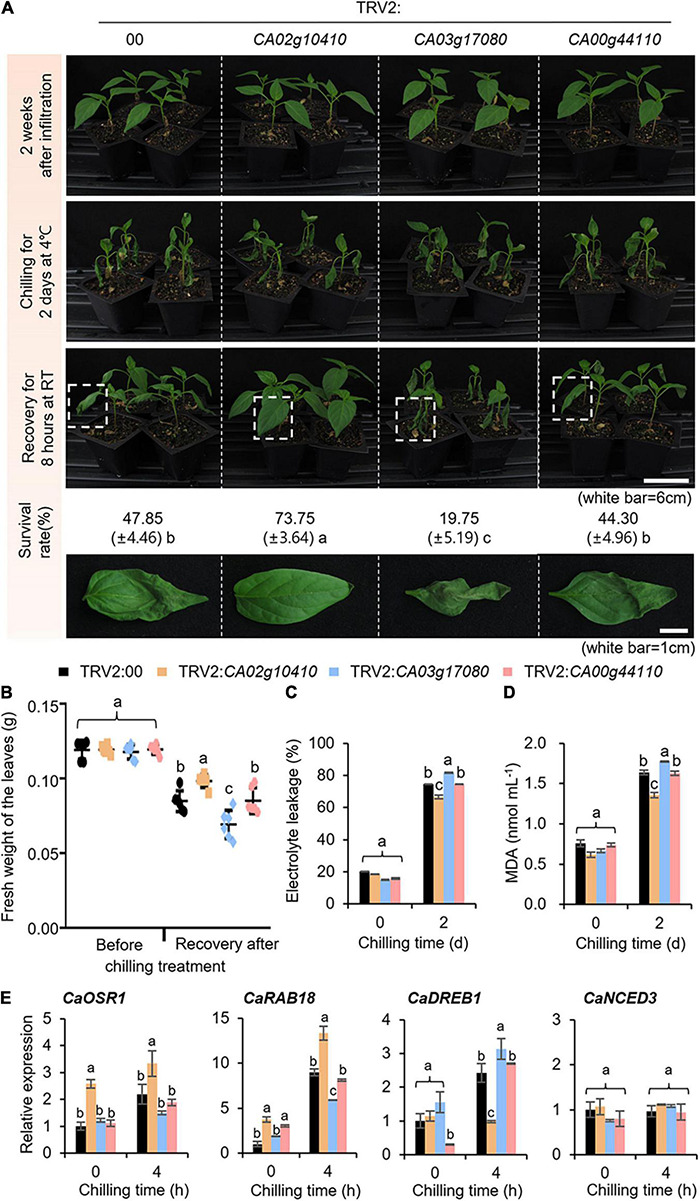
Phenotypic analysis of group II *CaSAP* genes to low temperature. **(A)** The low-temperature phenotypes of group II TRV2:*CaSAP*. Four-week-old plants of each line were subjected to the low temperature stress by chilling (4°C) for 2 days, followed by recovery for 8 h and the survival rates of each line were calculated. **(B)** The relative fresh weights of each line were calculated prior to chilling and after recovery. **(C,D)** Effect of low temperature stress on electrolyte leakage **(C)** and lipid peroxidation **(D)** in the leaves of TRV2:00 and group II TRV2:*CaSAP* pepper plants. Four-week-old plants were subjected to the low temperature (4°C) for 2 days, as shown in **(A)** (*n* = 20 plants of each line per replicate, 3 replicates). **(E)** Expression analysis of low temperature-inducible genes in the leaves of TRV2:00 and group II TRV2:*CaSAP* pepper plants. The relative expression (ΔΔCT) of *CaSAP* was normalized to that of *CaACT1*, which was used as an internal control gene. All values are presented as the mean ± standard error of values obtained from three independent experiments. Different letters indicate significant differences among the control and TRV2:*CaSAP* pepper plants (ANOVA; *P* < 0.05).

**FIGURE 5:**
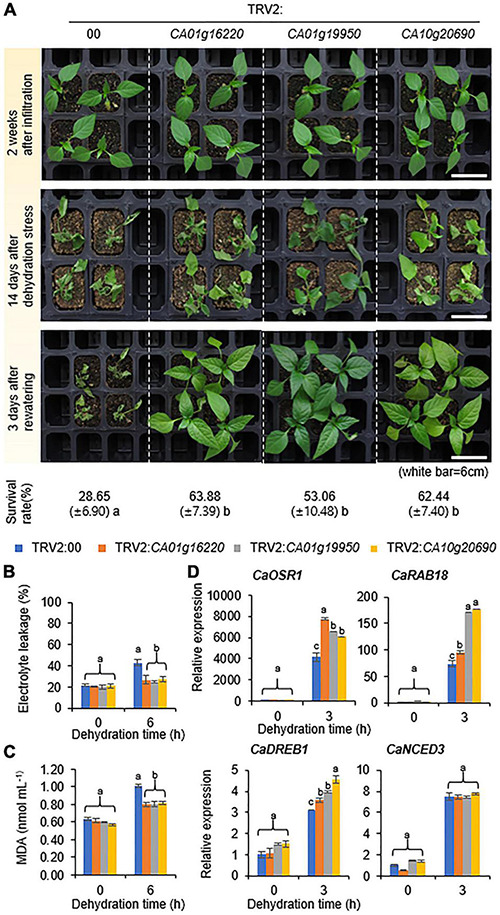
Phenotypic analysis of group IV *CaSAP* genes to the dehydration stress. **(A)** Dehydration-tolerant phenotypes of plants transformed with group IV TRV2:*CaSAP.* Four-week-old plants of each line were subjected to the dehydration stress by withholding watering for 14 days followed re-watering for 3 days. The percentages of surviving plants were calculated after re-watering. **(B,C)** Effect of dehydration stress on electrolyte leakage **(B)** and lipid peroxidation **(C)** in the leaves of TRV2:00 and group IV TRV2:*CaSAP* pepper plants. Four-week-old plants were subjected to the dehydration stress by detaching shoot for 6 h (*n* = 20 plants of each line per replicate, 3 replicates). **(D)** Expression analysis of dehydration-inducible genes in the leaves of TRV2:00 and group IV TRV2:*CaSAP* pepper plants. The relative expression (ΔΔCT) of *CaSAP* was normalized to that of *CaACT1*, which was used as an internal control gene. All values are presented as the mean ± standard error of values from three independent experiments. Different letters indicate significant differences among control and TRV2:*CaSAP* pepper plants (ANOVA; *P* < 0.05).

The expression of *CaSAP* genes was induced by subjecting plants to water stress caused by low temperature, dehydration, and osmotic stress (Figure 2); then, we examined the stress-related functions of these genes using a tobacco rattle virus-based virus-induced gene silencing (VIGS) system (Figures 4, 5). To verify the efficiency of VIGS, we performed RT-PCR analysis (Supplementary Figure 1) and established that the levels of *CaSAP* gene expression were lower in *CaSAP*-silenced pepper plants than in control plants. Under normal growth conditions, we were unable to detect any phenotypic differences between the control and *CaSAP*-silenced pepper plants (Figures 4, 5). For group II *CaSAP* genes, we investigated the response to low temperature (Figure 4) by placing *CaSAP*-silenced and control (TRV2:00) pepper plants in a cold chamber at 4°C for 2 days and thereafter allowing the plants to recover at 25°C for 8 h (Figure 4A). Under low temperature conditions, we observed a shrinkage of the first and second leaves of both control and *CaSAP*-silenced pepper plants (Figure 4A, middle panel). During the recovery phase under normal growth conditions, we noted that recovery status of the different *CaSAP*-silenced pepper plants differed from that of the control plants. For example, while the recovery of *CA02g10410*-silenced pepper plants was more evident than that of the control plants, recovery of the *CA03g17080*-silenced pepper was found to be less pronounced, and *CA00g44110*-silenced pepper showed no appreciable differences in revival compared with the control. To quantify these observations, we calculated the survival rate and fresh weights of the first and second leaves before and after recovery (Figures 4A,B). Prior to chilling treatment, we detected no significant differences between the *CaSAP*-silenced and control plants. Nevertheless, in line with our expectations, we found that the fresh weights of *CaSAP*-silenced pepper leaves differed significantly (*CA02g10410* and *CA03g17080*) or not (*CA00g44110*) from those of the controls post-recovery. Low temperature reduces the ability root water uptake and causes dehydration stress (Steponkus, 1984). This stress leads to increasing electrolyte leakage and lipid peroxidation, so we examine these physiological responses. As expected, electrolyte leakage and malondialdehyde (MDA) content showed significantly lower in *CA02g10410*-silenced pepper plants and significantly higher in *CA03g17080*-silenced pepper plants than that of control plants. *CA00g44110*-silenced pepper plants showed no differences (Figures 4C,D). To examine the corresponding expression patterns of stress-responsive genes, we conducted qRT-PCR analysis of control and *CaSAP*-silenced pepper plants (Figure 4E). The expression levels of *CaOSR1* and *CaRAB18* showed patterns consistent with the findings of previous phenotypic analysis. Among the *CaSAP*-silenced plants, expression levels of the two genes in TRV2:*CA02g10410* pepper were found to be significantly higher than those in control plants at 4 h after chilling treatment, whereas the transcript levels of TRV2:*CA03g17080* pepper were found to be lower than those in control plants in response to chilling treatment. Notably, however, the expression levels of *CaDREB1* showed no significant correlation with drought phenotypes. Under basal conditions, with the exception in TRV2:*CA00g44110* pepper plants, the expression levels of *CaDREB1* did not show significant differences under basal conditions, whereas the expression of TRV2:*CA02g10410* and TRV2:*CA03g17080* pepper was lower and higher, respectively, than that of the control plants after chilling treatment. Moreover, we detected no significant differences between control and *CaSAP*-silenced lines with respect to *CaNCED3* expression.

### Group IV *CaSAP*-Silenced Pepper Plants Confer Tolerance to the Dehydration Stress

To investigate the response to the dehydration stress, we subjected group IV *CaSAP*-silenced and control (TRV2:00) pepper plants to dehydration by withholding water for 14 days and thereafter re-watering for 3 days (Figure 5A). Notably, all group IV *CaSAP*-silenced pepper plants showed less wilted phenotypes than the control plants. To quantify these responses, we calculated survival rates; in line with our expectations, the survival rates of the group IV *CaSAP*-silenced pepper (63.88% ± 7.39%, 53.06% ± 10.48%, and 62.44% ± 7.40%) were found to be significantly higher than those of control plants (28.65% ± 6.90%). To identify whether these phenotypes were related to physiological responses, we examined electrolyte leakage and lipid peroxidation. For this, the shoots of group IV *CaSAP*-silenced and control pepper plants were detached and dehydrated for 6h. As expected, electrolyte leakage and malondialdehyde (MDA) content showed significantly lower in group IV *CaSAP*-silenced pepper plants than control (Figures 5B,C). To establish whether these tolerance phenotypes were associated with the expression levels of stress-responsive genes, we performed qRT-PCR as described in the previous section (Figure 5D and Supplementary Table 1). Under normal growth conditions, we detected no significant differences between control and *CaSAP*-silenced pepper plants with respect to stress-responsive gene expression. However, the expression levels of *CaOSR1*, *CaRAB18*, and *CaDREB1*, but not *CaNCED3*, were found to be higher in *CaSAP*-silenced pepper than in the control plants after 3 h of dehydration treatment. These findings provide evidence that group IV *CaSAP* genes play a negative functional role in the response of pepper to dehydration.

## Discussion

Stress-associated proteins zinc-finger proteins have been reported to be associated with the immune system in humans and multiple stress responses in plants (Evans et al., 2004; Chang et al., 2011; Giri et al., 2013; Zhang et al., 2019). To date, *SAP* genes have been identified in various monocot and dicot plant species, including *Arabidopsis thaliana*, *Oryza sativa*, *Solanum lycopersicum*, and *Medicago truncatula* (Vij and Tyagi, 2006; Solanke et al., 2009; Gimeno-Gilles et al., 2011; Gao et al., 2016; Dong et al., 2018; Saad et al., 2018, 2019; Xu et al., 2018; He et al., 2019; Li et al., 2019; Zhang et al., 2019; Lai et al., 2020; Wang et al., 2021). However, prior to the present study, there had been no similar characterization of *SAP* genes in *Capsicum annuum*.

In this study, we identified 14 *SAP* genes in *Capsicum annuum*. However, unlike our study, there is a wide variability for number of gene members in various plant such as 27 *SAP* genes in *Glycine max* and 57 genes in *Brassica napus* (He et al., 2019; Zhang et al., 2019). As suggested by previous studies (Gao et al., 2016; Dong et al., 2018; Lai et al., 2020), this phenomenon is possibly due to gene duplications. Consistently, we found 4 pairs of *CaSAP* genes have undergone dispersed and transposed gene duplication events in pepper plants (Supplementary Figure 2A).

In plants, *cis*-regulatory elements play an important role in the transcriptional regulation involved in growth, development, and different stress responses (Narusaka et al., 2003; Yamaguchi-Shinozaki and Shinozaki, 2005). In the present study, we found that the promoter regions of *CaSAP* genes are characterized by the presence of multiple types of these regulatory elements, including ABRE, MYB, MYC, STR, STRE, and MBS, which are associated with the responses to abiotic stresses (Table 2). In previous studies, it has been noted that a common feature of the *SAP* genes identified in different species is that a large proportion is intron-less genes. In rice, tomato, soybean, and castor bean, for example, a majority of the *SAP* genes lack introns, whereas a small number *SAP* genes have very few introns in their genomic information (Vij and Tyagi, 2006; Solanke et al., 2009; Zhang et al., 2019; Wang et al., 2021). Similarly, in pepper, type I CaSAPs, which contain A20 or AN1 domains, are lacking in introns, whereas the AN1 or C2H2 domain-contain type II CaSAPs are characterized by a single intron in their genomic region (Table 1 and Supplementary Figure 2B). Conceivably, by minimizing the number of steps necessary for post-transcriptional processing, this absence of intronic regions may confer the ability to mount a more rapid and precise response to stress stimuli (Jeffares et al., 2008; Grzybowska, 2012; Liu et al., 2021). These findings thus provide evidence indicating that *CaSAP* genes may play role in the rapid responses to different abiotic stresses.

In the present study, compared with the genes in other *CaSAP* groups, group II *CaSAP* genes were found to be highly induced following exposure to a low temperature (Figure 2). The *CA02g10410*-, *CA03g17080*-, and *CA00g44110*-silenced pepper plants showed different phenotypes under low-temperature conditions (Figure 4). Moreover, we observed that the expression of group IV *CaSAP*s was induced by drought stress and accordingly predicted that group IV *CaSAP*s would act as positive regulators of drought stress. However, contrary to our expectations, we found that pepper plants in which these genes had been knocked down displayed drought-tolerant phenotypes (Figure 5). There are two plausible explanations that could account for the observed phenotypes: (i) the induced genes may play a role in the defense response or contribute to the recovery of plants to normal growth and development; and (ii) gene transcription is regulated by multiple processes; hence, both positive and negative regulators may be induced by the same signal. In this context, the transcripts of group A PP2Cs have been shown to accumulate under drought stress conditions via a negative feedback regulatory loop (Merlot et al., 2001) and are induced by ABA and function as negative regulators of this phytohormone (Lim et al., 2015).

Abscisic acid is a major phytohormone that regulates seed germination, stomata closure, plant growth, and stress responses (Schroeder et al., 2001; Fujita et al., 2011; Nakashima and Yamaguchi-Shinozaki, 2013); previous studies have reported that several *SAP* genes, including *AtSAP13*, *AtSAP9*, *OsSAP1*, and *GmSAP16*, are induced by ABA and confer resistance to several abiotic stresses. In addition, *GmSAP16* and *AtSAP9* affect the expression levels of stress-related genes (Mukhopadhyay et al., 2004; Kang et al., 2017; Dixit et al., 2018; Zhang et al., 2019). Numerous stress-related genes associated with defense responses are induced when plants are exposed to different stress conditions (Gonzalez-Guzman et al., 2012; Park et al., 2015). For example, NCED3, induced by several sources of abiotic stress, is a key enzyme in ABA biosynthesis (Iuchi et al., 2001; Endo et al., 2008). In the present study, we found that three of the 14 identified *CaSAP* genes, all of which clustered in the group II category of *SAP*s, were significantly induced by exogenous ABA (Figure 2). Notably, in a manner similar to that reported in previous studies, we found that two of the group II *CaSAP* genes (*CA02g10410* and *CA03g17080*) had considerable effects on the expression of other stress-related genes, including *CaOSR1* (homologous to *RD29B*), *CaRAB18*, and *CaDREB1*, in response to the low temperature stress (Figure 4C). Moreover, whereas the group IV *CaSAP* genes were not induced by exogenous ABA, we found that they contributed to an increase in the expression levels of *CaOSR1*, *CaRAB18*, and *CaDREB1* in response to the dehydration stress (Figure 5B). In contrast, however, we detected no significant differences in the expression of *CaNCED3* between group II or IV *CaSAP*-silenced pepper and control plants (Figures 4C, 5B). These observations indicate that the group II and IV *CaSAP* genes may play a more weighted role in the stress response by binding to a specific site of the stress-responsive gene promoter rather than being involved in the ABA biosynthetic process.

## Conclusion

In conclusion, on the basis of our findings, we suggest that CaSAPs can function either positively or negatively in mediating abiotic stress responses in *Capsicum annuum*. We identified 14 *SAP* genes in pepper plants, which were characterized genomically. Moreover, we were able to classify these genes based on domain and phylogenetic analyses and provisionally characterize the functions of the encoded SAP proteins. However, we were unable to precisely elucidate CaSAP function, nor were we able to identify the downstream genes. Thus, in further studies, we intend to focus on the mechanisms underlying SAP-mediated stress response based on molecular approaches and seek to identify the corresponding target genes.

## Data Availability Statement

The original contributions presented in the study are included in the article/Supplementary Material, further inquiries can be directed to the corresponding author/s.

## Author Contributions

YB and CL performed the experiments and analyzed the results. SL designed the experiments. YB and SL wrote the manuscript. All the authors contributed to the article and approved the submitted version.

## Conflict of Interest

The authors declare that the research was conducted in the absence of any commercial or financial relationships that could be construed as a potential conflict of interest.

## Publisher’s Note

All claims expressed in this article are solely those of the authors and do not necessarily represent those of their affiliated organizations, or those of the publisher, the editors and the reviewers. Any product that may be evaluated in this article, or claim that may be made by its manufacturer, is not guaranteed or endorsed by the publisher.

## Footnotes

1
https://vigs.solgenomics.net/

2
https://solgenomics.net/

3
https://www.dna.affrc.go.jp/PLACE/?action=newplace
