# Supplementary material for: Differential Functions of Pepper Stress-Associated Proteins in Response to Abiotic Stresses
Source: Front Plant Sci. 2021 Dec 10;12:756068. doi: 10.3389/fpls.2021.756068 (PMC8702622; doi:10.3389/fpls.2021.756068)
Supplement: Supplementary file 1 [file Table_1.pdf]

Supplementary Table 1. List of primers used in this study.

Name		sequence (5'-3')
**For cloning**		
CA02g10410 CDS	Forward	ATGACTTCTTGTAACAAAGTATTAGTAACT
	Reverse (w/o stop codon)	GATCCTATTCTCAAGTTTGTCAGCTT
CA03g17080 CDS	Forward	ATGACTTCAGTAATAACCCCAATTAGTA
	Reverse (w/o stop codon)	GATCCTATTCTCAAGTTTATCACCTTT
CA00g44110 CDS	Forward	ATGAGTTCTTGTTACAAAGCAGAAAAA
	Reverse (w/o stop codon)	GATCCTAGTCTCAAGTTTATCAACTTTG
CA01g16220 CDS	Forward	ATGGAGGCATGGTGTGTTGACAATA
	Reverse (w/o stop codon)	GATCTTGTCAAGTTTTTCTGCTTTAAC
CA01g19950 CDS	Forward	ATGGAGCAAAATGATACAGGCTG
	Reverse (w/o stop codon)	TATTTTGTCAAGCTTTTCAGCCTTA
CA10g20690 CDS	Forward	ATGGAGTCTTCCAAAGAGACAGGC
	Reverse (w/o stop codon)	GATCTTGTTTAGCTTTTCCGCTAC
**For VIGS**		
CA02g10410 VIGS	Forward	TCTAGAATGACTTCTTGTAACAAAGTATTA
	Reverse	CTCGAGATATCTATGACTTATGCAAAA
CA03g17080 VIGS	Forward	TCTAGAATGACTTCAGTAATAACCCCA
	Reverse	CTCGAGAGCACATGCATGTTCTTC
CA00g44110 VIGS	Forward	TCTAGAATGAGTTCTTGTTACAAAGCAG
	Reverse	CTCGAGCATTCCTCCTCCACATC
CA01g16220 VIGS	Forward	TCTAGATGTGTTGACAATATGGAGCAT
	Reverse	CTCGAGGCTTGAAGTTTGAGATGATGGCAAAG
CA01g19950 VIGS	Forward	TCTAGAATGGAGCAAAATGATACAGGCTGCCAA
	Reverse	CTCGAGTTCTTCGCTCTCGGGTGGACATGCAA
CA10g20690 VIGS	Forward	TCTAGAATGGAGTCTTCCAAAGAGACA
	Reverse	CTCGAGTTGACCTGAGGATAAAGC
**For RT-PCR**		
CA01g04040 RT	Forward	GGACTTTGTTCCAAGTGTTACAGAG
	Reverse	ACATCTGCACTTAAATCCCACTACT
CA02g10410 RT	Forward	CTCCAATTTCATGCGAGAGTAGTTG
	Reverse	ACAAATGAACCTTGTTAATCCCACC
CA03g17080 RT	Forward	CCCAATTAGTAGTTGTGGCTTTTGT
	Reverse	TATTAATCCCACCTTCTTCCTGCAA
CA00g44110 RT	Forward	TTATGTTCAAGGGGTTGTGGTTTTT
	Reverse	CATCGACAGCTAAACCCTGTTAATC
CA10g16510 RT	Forward	CGATACTATCTTTCCTCCTCCACAG
	Reverse	TGAAATCAAACGAACATCCGTGAAT
CA12g06430 RT	Forward	CATTTTGTGGTTCACATAGGTACCC
	Reverse	ATCATCCATGATCATCACCAAAACG
CA01g16220 RT	Forward	CATGTGCTCCAAGTGTTACAAAGAT
	Reverse	TAGGACCCACTTTAGCCTTTACTTC
CA01g19950 RT	Forward	GAGCAAAGAAGTTGAGAAGGCTAAG
	Reverse	CAGCTCATAACTCGAACATTCCATC
CA10g20690 RT	Forward	TGTAAATGGAAACTCAAGCAGCAAT
	Reverse	ATCCCGACCAGCATTCTTATAATCA
CA01g19980 RT	Forward	CTGGAAAGGATGAGTCAAGATCTGA
	Reverse	GCGATTTTCGTGTTTTCCTTTTCAA
CA12g22410 RT	Forward	GCACCGTAACAAATAACGATCTGAT
	Reverse	TGGGAGCTTTAACAACTGGATTTTC
CA02g29220 RT	Forward	CACTGCCAACTATCTGATTGTCATC
	Reverse	CCAGATTTCTCGTGTTTCTGCAATA
CA05g01100 RT	Forward	AGTGAGTTTAACCAAACACAGCAAA
	Reverse	TGTTCCTTTTCAACATGTTCCACAA
CA10g19590 RT	Forward	ATGGGTACACCAGAATTCCCAAAT
	Reverse	TTTCGTAGTTTGATGGATCACACTCG
CaACT1 RT	Forward	GACGTGACCTAACTGATAACCTGAT
(CA12g08730)	Reverse	CTCTCAGCACCAATGGTAATAACTT
CaOSR1 RT	Forward	ATGGAGGCACAACTGCACCGTC
(CA03g17780)	Reverse	GGCCCACCATGAACTTCTGCAC
CaRAB18 RT	Forward	ATGTCGCACTACGAGAACCAATATAG
(CA02g22060)	Reverse	ATCATCCTCAGAGCTGCTGGAGC
CaNCED3 RT	Forward	TTAAGGATCTTAAGCGTGGTTATGT
(CA08g03620)	Reverse	AGATTAGTTCAAGAACGTGAATTGG
CaDREB1 RT	Forward	GGAATTTCAAAAGAATCATCTAGCA
(CA03g16520)	Reverse	AGCTAGAAGAGTACTGCATCCAAAA
