# Supplementary material for: Differential Functions of Pepper Stress-Associated Proteins in Response to Abiotic Stresses
Source: Front Plant Sci. 2021 Dec 10;12:756068. doi: 10.3389/fpls.2021.756068 (PMC8702622; doi:10.3389/fpls.2021.756068)
Supplement: Supplementary file 2 [file Image_1.PDF]

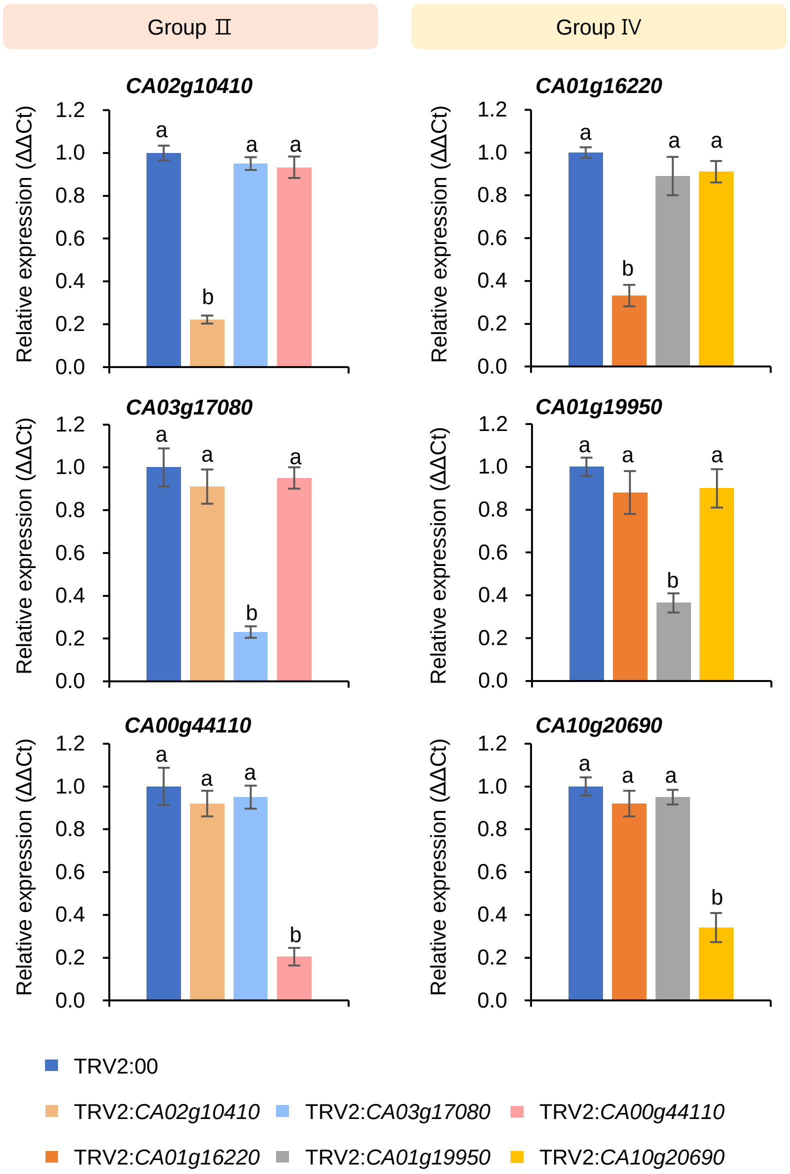

Supplementary Figure 1. qRT-PCR analysis of the expression of group II and IV *CaSAP* genes following virus-induced gene silencing. Expression levels of group II and IV *CaSAP* genes were determined based on qRT-PCR analysis using cDNA derived from the first and second leaves of pepper plants transfected with an empty vector control (TRV2:00) or *CaSAP*-silenced constructs (TRV2:*CA01g16220*, TRV2:*CA01g19950*, TRV2:*CA10g20690*, TRV2:*CA02g10410*, TRV2:*CA03g17080*, and TRV2:*CA00g44110*). *CaACT1* was used as an internal control gene.
