# Supplementary material for: Differential Functions of Pepper Stress-Associated Proteins in Response to Abiotic Stresses
Source: Front Plant Sci. 2021 Dec 10;12:756068. doi: 10.3389/fpls.2021.756068 (PMC8702622; doi:10.3389/fpls.2021.756068)
Supplement: Supplementary file 3 [file Image_2.PDF]

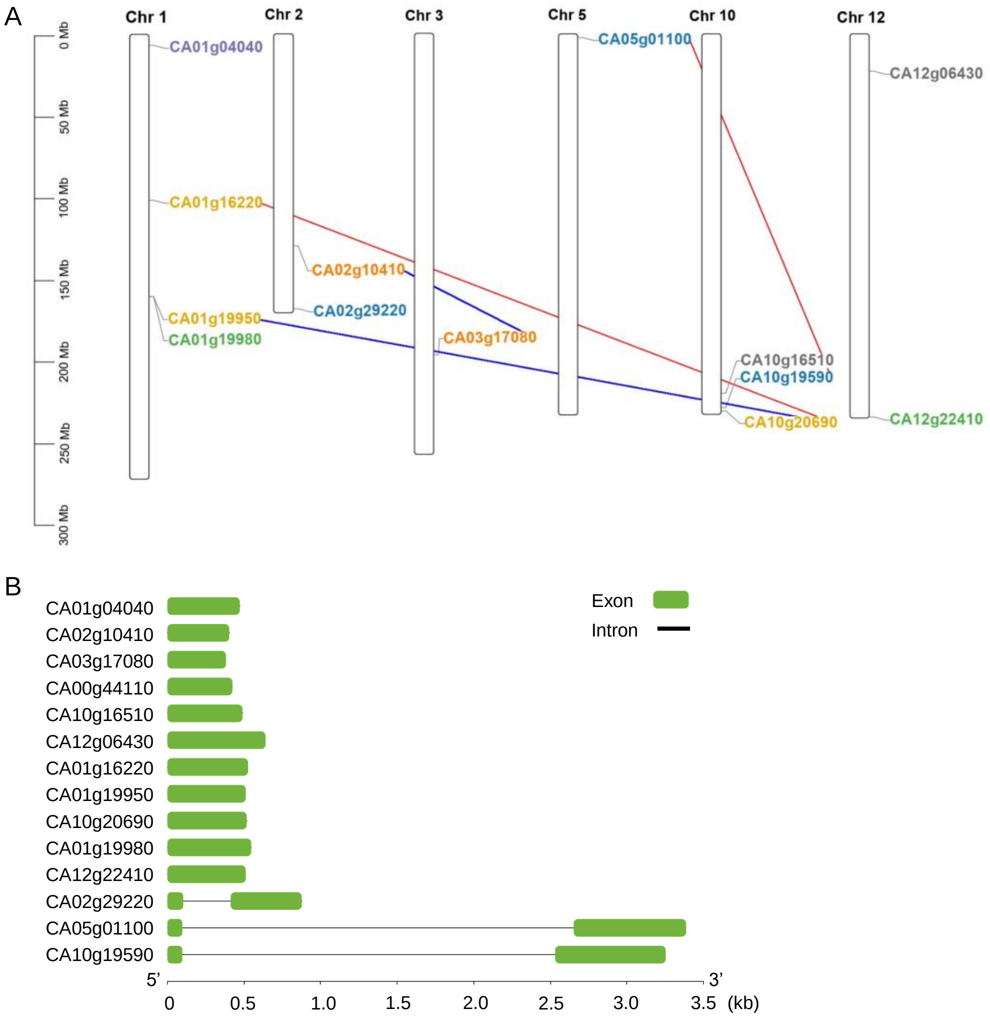

Supplementary Figure 2. The pepper SAP family. (A) Chromosomal location of *CaSAP* genes. Each gene position within the 12 pepper chromosomes were drawn by Tbtools (Chen et al., 2020). Duplication events of *CaSAP* genes were identified using Plant duplicate gene database (PlantDGD; http://pdgd.njau.edu.cn:8080/). Red line indicates the dispersed duplication and blue line indicates the transposed duplication. (B) Exon-intron structures of *CaSAP* genes. Green boxes represent exons and black lines indicate introns.
